# Temporary hypocalcemia induced by cetuximab sarotalocan without hypomagnesemia in a patient with hypoparathyroidism: a novel case report

**DOI:** 10.1186/s40780-025-00465-y

**Published:** 2025-07-04

**Authors:** Moeko Kado, Yoshitaka Saito, Tatsuhiko Sakamoto, Takayoshi Suzuki, Yoh Takekuma, Mitsuru Sugawara

**Affiliations:** 1 Department of Pharmacy, Hokkaido University Hospital, Kita 14-Jo, Nishi 5-Chome, Kita-Ku, Sapporo, 060-8648 Japan; 2 Department of Clinical Pharmaceutics & Therapeutics, Faculty of Pharmaceutical Sciences, Hokkaido University of Science, 4-1, Maeda 7-Jo 15-Chome, Teine-Ku, Sapporo, 006-8585 Japan; 3 Department of Otolaryngology-Head and Neck Surgery, Faculty of Medicine and Graduate School of Medicine, Hokkaido University, Kita 15-Jo, Nishi 7-Chome, Kita-Ku, Sapporo, 060-8638 Japan; 4 Department of Forensic Medicine, Faculty of Medicine and Graduate School of Medicine, Hokkaido University, Kita 15-Jo, Nishi 7-Chome, Kita-Ku, Sapporo, 060-8638 Japan; 5 Laboratory of Pharmacokinetics, Faculty of Pharmaceutical Sciences, Hokkaido University, Kita 12-Jo, Nishi 6-Chome, Kita-Ku, Sapporo, 060-0812 Japan

**Keywords:** Hypocalcemia, Cetuximab sarotalocan, Hypomagnesemia, Hypoparathyroidism, Hypokalemia, Epidermal growth factor receptor

## Abstract

**Background:**

Cetuximab sarotalocan, which utilizes the light-activatable dye IRDye700Dx conjugated with cetuximab, is a first-in-class drug based on photoimmunotherapy for treating recurrent head and neck squamous cell carcinoma. Cetuximab frequently induces hypomagnesemia and secondary hypocalcemia. Herein, we report a case of independent hypocalcemia without hypomagnesemia during treatment and discuss symptom progression.

**Case Presentation:**

A female patient with left epipharyngeal cancer, hypothyroidism, and hypoparathyroidism was treated with cetuximab and sarotalocan. On day 3, serum-adjusted calcium levels decreased from 9.6 to 7.4 mg/dL, increased to 8.2 mg/dL on day 9, and further increased to 8.8 mg/dL on day 27; serum magnesium levels were not evaluated. After the second administration, serum-adjusted calcium levels decreased two days later, fluctuating between 7.6 and 8.1 mg/dL for three weeks. Serum magnesium levels were within the normal range, with no significant variation detected during the second cycle. A similar symptom course was observed during the third cycle. The patient received enteral nutrition daily with 424.8–1,038.4 mg of calcium, with daily adjustment during the administration, except on day 2. She received peripheral intravenous nutrition for several days after tumor illumination. Concomitant medications did not appear to affect serum calcium levels. Considering the case process and previous reports, we hypothesized that concomitant hypoparathyroidism, in addition to reduced calcium intake due to the treatment, may have contributed to the observed reduction.

**Conclusions:**

Hypocalcemia without hypomagnesemia can occur in patients with hypoparathyroidism receiving near-infrared photoimmunotherapy with cetuximab sarotalocan. The precise mechanisms and epidemiological features warrant further investigations.

**Supplementary Information:**

The online version contains supplementary material available at 10.1186/s40780-025-00465-y.

## Background

Head and neck cancer is the seventh most common cancer worldwide, accounting for nearly 900,000 new diagnoses annually [1, 2]. Head and neck squamous cell carcinoma (HNSCC) accounts for 90% of head and neck cancers, and patients with progressive or recurrent HNSCC have limited treatment options [3, 4].

Near-infrared photoimmunotherapy (NIR-PIT) is a photo-activated cancer therapy based on the photo-induced ligand release reaction. This emerging treatment modality utilizes a light-activatable dye IRDye700Dx (IR700) conjugated to a monoclonal antibody targeted against tumor-associated antigens located on the tumor cell surface [5].

Cetuximab sarotalocan is a first-in-class drug based on photoimmunotherapy for treating recurrent HNSCC [5–7]. This formulation comprises IR700 conjugated to cetuximab, a monoclonal antibody targeting the epidermal growth factor receptor (EGFR) [7]. Cetuximab sarotalocan photoimmunotherapy requires two steps to be conducted sequentially: (1) intravenous infusion over 2 h and (2) tumor illumination with nonthermal red light (690 nm) 24 ± 4 h after infusion [6, 7]. Given that anti-EGFR monoclonal antibodies decrease the stimulation of the transient receptor potential subfamily melastatin (TRPM) 6 in the distal convoluted tubule and inhibit TRPM6 channels in the gut, hypomagnesemia frequently occurs owing to reduced renal magnesium reabsorption and intestinal absorption [8]. Additionally, hypomagnesemia associated with anti-EGFR monoclonal antibody therapy occasionally leads to secondary hypocalcemia and hypokalemia [9].

Herein, we report a case of independent temporary hypocalcemia in addition to hypomagnesemia during cetuximab sarotalocan photoimmunotherapy and discuss the progression of symptoms.

## Case Presentation

A female patient was diagnosed with left epipharyngeal cancer (stage IIb) and had undergone excision with adjuvant radiation (58.4 Gy/31 fr) nineteen years ago. Eight years later, recurrence was detected in the anterior wall of the left maxilla, and partial resection was performed, followed by radiation (60 Gy/33 fr). After seven years, recurrence was observed in the palate, and partial resection was performed. Two years later, further recurrence was observed at the base of the nose, and partial resection was performed. After another two years, the cancer recurred in the upper jaw. Consequently, the patient (in her 50 s) was admitted to Hokkaido University Hospital for treatment with cetuximab sarotalocan.

She had developed hypothyroidism due to subtotal thyroidectomy 30 years ago, which was well managed with levothyroxine sodium. She was taking alfacalcidol to address hypoparathyroidism. In addition, she was diagnosed with breast cancer (stage 0, hormone receptor-positive) and underwent surgery followed by adjuvant treatment (cyclophosphamide + docetaxel followed by tamoxifen) seven years ago. Furthermore, she had developed chronic glomerulonephritis (chronic kidney disease grade 3aA1) six years ago, necessitating regularly administered darbepoetin alfa. Baseline systemic medication was oral levothyroxine sodium 75 µg/day and alfacalcidol 1 µg/day, with the same dosage administered for more than seven years, and subcutaneous darbepoetin alfa 60‒120 µg every ~ 8 weeks for 3 years.

On the day of treatment, the patient received intravenous 640 mg/m^2^ of cetuximab sarotalocan for 2 h. To prevent photosensitivity, the brightness in the room was set to < 120 lx for 7 days after administration. Laser irradiation was performed 22–26 h after the end of cetuximab sarotalocan administration (24 h for the first administration and 22 and 26 h for the second and third administrations, respectively). Table 1 summarizes the laboratory data before administration. Serum-adjusted calcium, magnesium, potassium, sodium, and phosphorus levels were 9.6 mg/dL, 1.8 mg/dL, 4.1 mEq/L, 141 mEq/L, and 3.9 mg/dL, respectively, which were all within the normal range without notable variation during approximately one year prior to the administration. Baseline creatinine clearance calculated using the Cockcroft-Gault formula was 48.4 mL/min.

**Table 1:** Laboratory data before the first administration

Calcium (mg/dL)	9.6
Magnesium (mg/dL)	1.8
Potassium (mEq/L)	4.1
Sodium (mEq/L)	141
Chloride (mEq/L)	102
Phosphorus (mg/dL)	3.9
Creatinine (mg/dL)	0.98
Creatinine clearance (mL/min)	48.4
Blood urea nitrogen (mg/dL)	25
Free T4 (ng/dL)	1.26
TSH (mIU/L)	2.34
Albumin (g/dL)	4.3
AST (U/L)	25
ALT (U/L)	17
γ-GT (U/L)	22
CRP (mg/dL)	< 0.02

*γ-GT* γ-glutamyltransferase, *ALT* Alanine transaminase, *AST* Aspartate transaminase, *CRP* C-reactive protein, *TSH* Thyroid stimulating hormone

**Fig. 1:**
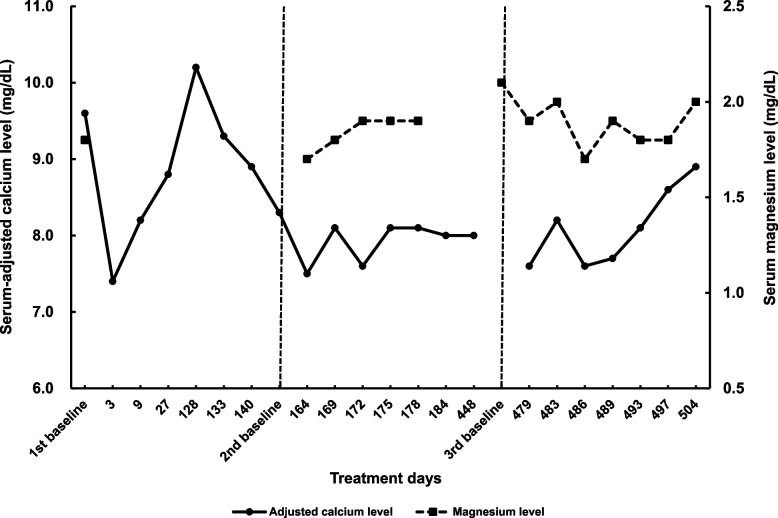
Variations in serum-adjusted calcium and magnesium levels. The first dose of cetuximab sarotalocan was administered on day 1, with second and third doses administered on days 162 and 477, respectively

**Fig. 2:**
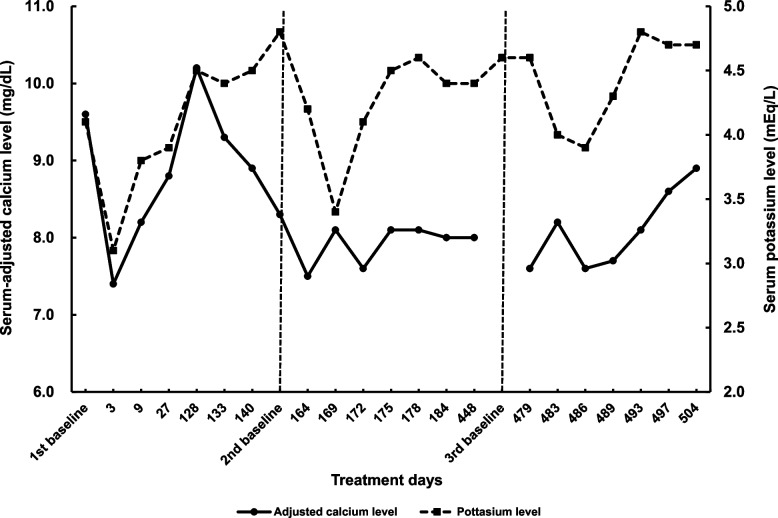
Variation of adjusted calcium and potassium levels. The first dose of cetuximab sarotalocan was administered on day 1. The second and third doses were administered on days 162 and 477, respectively

Figures 1 and 2 illustrate variations in serum electrolyte levels. On day 3, serum-adjusted calcium levels decreased from 9.6 to 7.4 mg/dL, and serum potassium levels decreased from 4.1 to 3.1 mEq/L. The pharmacist consulted a physician regarding the need for calcium supplementation. After discussion, we decided to closely monitor the patient’s condition without supplementation, while the serum calcium levels remained low, as no neurological nor gastrointestinal symptoms were observed. On day 9, the serum-adjusted calcium level increased to 8.2 mg/dL, along with an elevation in the serum potassium level. On day 27, the serum-adjusted calcium level further increased to 8.8 mg/dL, while the serum potassium level remained stable. Unfortunately, serum magnesium levels were not evaluated during the first administration. On day 128, the serum-adjusted calcium level was elevated to 10.2 mg/dL, leading to the dose reduction of alfacalcidol to 0.5 µg/day on day 133 and further decrease to 0.25 µg/day on day 140. Accordingly, the serum-adjusted calcium level decreased to 8.3 mg/dL at the baseline of the second cycle. On day 162, a second dose of cetuximab sarotalocan was administered. Since a decrease in serum calcium levels was anticipated in the second cycle, as in the first cycle, the pharmacist proactively requested that the physician order a blood test that included serum calcium and other electrolyte analyses. Two days later (day 164), the serum-adjusted calcium level was 7.5 mg/dL, fluctuating between 7.6 and 8.1 mg/dL during the following three weeks (until day 184). The hypocalcemia symptoms were monitored as in the first cycle. Serum potassium level decreased to 3.4 mEq/L from 4.8 mEq/L seven days after the administration (day 169), although it increased to 4.1 mEq/L three days later (day 172). Serum magnesium levels were within the normal range, and no significant variations were observed during the three weeks of the second cycle. The third dose was administered on day 477. The serum-adjusted calcium level reached 7.6 mg/dL two days after administration, increasing to 8.6 mg/dL 20 days after the administration (day 497). Similarly, in the third cycle, the pharmacist requested that the physician perform blood tests and continue monitoring for symptoms of hypocalcemia. Serum potassium levels also showed a temporary decrease between days 483 and 489 but increased to baseline levels on day 493. In this treatment cycle, the serum magnesium level was fully assessed and remained stable within the normal range during treatment. Serum sodium levels were stable within 134‒142 mEq/L, and serum creatinine levels were stable between 0.6 and 0.8 mg/dL during the treatment cycles (detailed data not shown).

**Fig. 3:**
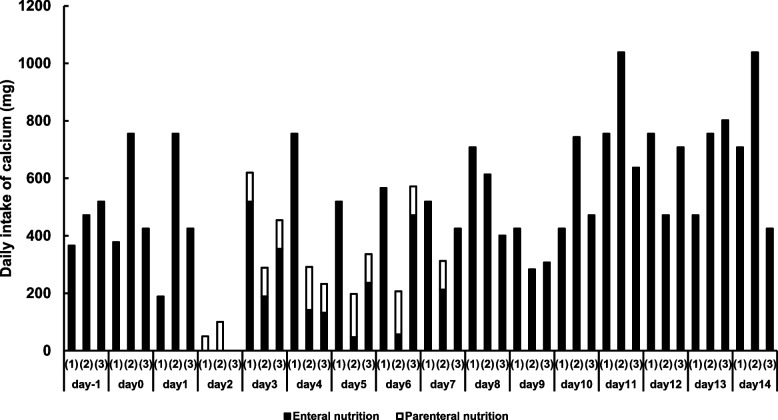
Variation of calcium intake. The numbers in parentheses indicate the number of treatment cycles

The patient had a gastrostomy tube inserted three years ago due to swallowing difficulties post-surgery and received enteral nutrition with daily 900–2,200 kcal and 424.8–1,038.4 mg of calcium, with daily adjustment during the administration of cetuximab sarotalocan, except on day 2. Oral intake of fluids was performed using a thickener according to the patient's wishes. The patient fasted on day 2 and received peripheral intravenous nutrition (BFLUID® injection, 2.5 mEq of Ca^2+^ per 500 mL) from day 2 or 3 for 2–6 days, depending on the amount of enteral nutrition (Fig. 3). Moreover, the patient did not experience adverse effects such as vomiting or diarrhea, which can reduce serum calcium or potassium levels during treatment.

Concomitant medications taken during treatment are shown in Table 2. Intravenous or transdermal fentanyl, intravenous or oral acetaminophen, epidural levobupivacaine, intravenous flurbiprofen axetil, and oral loxoprofen were administered to control postoperative pain. Intravenous cefazolin or oral amoxicillin was administered to prevent or treat infection, and oral minocycline was temporarily administered for rash prophylaxis. During the treatment course, medications were also administered for managing suspicious infusion-related reactions (hydrocortisone), defecation control (*Clostridium butyricum* and naldemedine), insomnia (lemborexant), and gastrointestinal disorder (lansoprazole). As needed, medications, such as magnesium oxide, sodium picosulfate, a sodium bicarbonate suppository, oxycodone, and metoclopramide, were administered to temporarily treat patient discomfort.

**Table 2:** Systemic medication during the treatment

Baseline
Oral levothyroxine sodium 75 µg once daily
Oral alfacalcidol 1 µg once daily
Subcutaneous darbepoetin alfa 60–120 µg administered approximately every 8 weeks
After the first cycle
Days 0–26 intravenous fentanyl 200–1,500 µg/day, followed by fentanyl tape 1 mg daily from days 27–125
Days 1–8 oral minocycline 50 mg twice daily
Days 2–3 intravenous cefazolin 1 g once daily
Days 8–22 oral acetaminophen 800 mg thrice daily
Day 10 subcutaneous darbepoetin alfa 60 µg
Between the first and second cycle
Day 70 subcutaneous darbepoetin alfa 60 µg
Days 126–139 fentanyl tape 0.5 mg daily
Day 128 subcutaneous darbepoetin alfa 120 µg
Days 133–139 oral alfacalcidol 0.5 µg once daily
Day 140 oral alfacalcidol 0.25 µg once daily
After the second cycle
Days 162–169 intravenous fentanyl 300–1,750 µg/day, followed by fentanyl tape 3 mg from days 170–171, 2 mg from days 172–173, 1 mg from days 174–175, and 0.5 mg from days 176–178
Days 162–168 oral minocycline 50 mg twice daily
Day 163 intravenous cefazolin 1 g twice a day
Day 163 intravenous hydrocortisone 500 mg once a day
Day 167–177 oral naldemedine 0.2 mg once a day
Day 170–476 oral acetaminophen 800 mg thrice a day
Day 178–206 oral Clostridium butyricum 1 g thrice a day
Between the second and third cycle
Day 184, 240, 289, 345, 401, and 457 subcutaneous darbepoetin alfa 120 µg
After the third cycle
Days 477–488 intravenous fentanyl 300–2,250 µg/day; this was followed by fentanyl tape 5 mg from days 489–493, 4 mg from days 494–498, 3 mg from days 499–502, 2 mg from days 503–506, and 1 mg from days 507–514
Day 477–490 oral minocycline 50 mg twice daily
Day 477–483 intravenous flurbiprofen axetil 50 mg thrice daily, followed by oral loxoprofen 60 mg thrice daily
Day 477–483 intravenous acetaminophen 800 mg four times daily, followed by oral acetaminophen 800 mg four times daily from days 484‒582
Day 478 intravenous cefazolin 1 g twice daily
Day 478 intravenous hydrocortisone 200 mg once daily
Day 478 epidural levobupivacaine 75 mg once daily
Days 478–515 naldemedine 0.2 mg once daily
Day 484–582 oral lansoprazole 30 mg once daily
Days 485–487 intravenous cefazolin 1 g four times daily, followed by oral amoxicillin 250 mg thrice daily from days 491–505
Day 486 oral *Clostridium butyricum* 1 g thrice daily
Day 501 oral lemborexant 5 mg once daily

## Discussion and Conclusions

Hypocalcemia is one of the most frequent electrolyte disorders associated with anti-EGFR monoclonal antibody therapy. According to a pooled analysis, all-grade hypocalcemia caused by cetuximab was observed in 16.8% of cases, and grade 3/4 symptoms induced by either cetuximab or panitumumab were reported in 3.8% of all cases [10]. The general mechanisms of anti-EGFR treatment-induced hypocalcemia are multifactorial and associated with hypomagnesemia [9]. Impaired magnesium-dependent adenyl cyclase generation by cAMP reduces the release of parathyroid hormone, and skeletal resistance to this hormone during magnesium deficiency has also been implicated [9]. Additionally, hypomagnesemia alters the normal heteroionic exchange of calcium and magnesium at the bone surface, leading to increased bone release of magnesium ions in exchange for increased skeletal uptake of calcium from the serum [9]. Thus, hypocalcemia during anti-EGFR therapy is considered secondary to hypomagnesemia. Maliakal et al. suggested that hypocalcemia is a classic sign of severe hypomagnesemia (< 1.2 mg/dL) [9]. However, in the current case, we observed independent hypocalcemia without hypomagnesemia or notable serum magnesium variation, considering the electrolyte changes during each treatment cycle; however, several laboratory data were missing. The concomitant medications described in Table 1, except lansoprazole, were not associated with hypocalcemia, and lansoprazole was administered during the third cycle alone. Thus, we considered the symptoms observed in the current case were due to cetuximab sarotalocan and assessed the relationship using the Naranjo Adverse Drug Reaction Probability Scale, with a score of 6, indicating a"Probable"association (Supplemental Table 1).

Although there have been previous reports of electrolyte abnormalities associated with cetuximab sarotalocan, hypocalcemia has not been reported [11]. Furthermore, it remains unclear whether the reported electrolyte abnormalities are attributable to cetuximab or sarotalocan components. Although hypocalcemia has been reported with cetuximab alone, no such reports exist for cetuximab sarotalocan; therefore, we hypothesized that cetuximab caused the observed hypocalcemia.

Interestingly, the reduced serum potassium levels occurred in parallel with serum calcium levels, whereas serum sodium and chloride levels did not. In addition, a decrease in serum potassium levels can be occasionally observed in patients administered anti-EGFR monoclonal antibodies, typically secondary to hypomagnesemia [9]. However, the findings in our case differed, suggesting the occurrence of independent hypocalcemia and hypokalemia apart from hypomagnesemia.

Thomas et al. have reported a similar case of independent hypocalcemia associated with cetuximab; a patient post parathyroidectomy without renal impairment received cetuximab and irinotecan for metastatic colorectal cancer therapy and exhibited severe hypocalcemia after one week of chemotherapy (decrease from 7.0 to 5.7 mg/dL) [12]. She re-developed symptoms despite receiving oral calcium carbonate 1,000 mg TID and magnesium oxide 420 mg BID. Impressively, a 24-h urine evaluation revealed low calcium excretion and elevated fractional excretion of magnesium, although the serum magnesium levels were within the normal range [12]. Moreover, Chen et al. reported two cases of cetuximab-induced refractory hypokalemia without hypomagnesemia [13]. A patient who underwent a urine examination exhibited a non-obvious increase in potassium excretion, similar to the hypocalcemia reported previously [12]. Based on these facts, the temporary decrease in serum calcium levels in the current case may be due to reduced calcium absorption and/or abnormalities in bone metabolism and not due to enhanced excretion; however, renal calcium excretion was not assessed in our patient.

This patient had developed hypoparathyroidism upon undergoing subtotal thyroidectomy, and substantially reduced serum calcium levels and hypocalcemia were observed after the first administration. The dosage of alfacalcidol was reduced on days 133 and 140, and serum calcium levels decreased after dose reduction, regardless of cetuximab sarotalocan administration. Additionally, calcium intake, particularly on day 2, was lower than usual in the first cycle, during which a notable decrease in serum calcium levels occurred. These results suggest that calcium intake possibly impacted serum calcium reduction after administration in the current case; however, it remains unclear whether reduced intake due to invasive tumor illumination or uptake inhibition by cetuximab sarotalocan influenced these results. Moreover, the lower serum calcium variations observed in the second and third cycles could be attributed to the lower impact of calcium intake owing to reduced alfacalcidol dosage, resulting in its reduced uptake.

Hypocalcemia is a typical symptom of hypoparathyroidism [14]. Serum calcium levels decrease 4 h after thyroidectomy, suggesting that calcium levels decrease rapidly without supplementation [15]. A previous case report documented the same symptoms with cetuximab treatment, with the patient having previously undergone parathyroidectomy [12]. Consequently, concomitant hypoparathyroidism, in addition to reduced calcium intake, may have contributed to the observed decrease in this patient (Fig. 1, Fig. 2), as it is unlikely that a substantial decrease in serum calcium levels occurred due to reduced calcium intake for a few days (Fig. 3) alone, from a homeostasis perspective. Furthermore, the patient exhibited moderate renal impairment, which may have affected the results, although the renal involvement may have been low.

When administering anti-EGFR monoclonal antibodies to patients with hypoparathyroidism, pharmacists should proactively request serum electrolyte tests, including calcium levels, to enable the early detection of hypocalcemia. It is also important to educate patients about the possible symptoms and potential delays in calcium recovery. Additionally, assessing total calcium intake from medications, enteral nutrition, and diet is a key aspect of pharmaceutical care.

In general, intravenous or oral calcium supplementation and adjustment of serum magnesium levels are performed to treat anti-EGFR monoclonal antibody-induced hypocalcemia [9, 16]. Active vitamin D supplementation is pivotal for managing chronic symptoms in patients with hypoparathyroidism [16]. In the current case, grade 2 hypocalcemia disappeared without medication, possibly due to a single administration of the suspected agent during a certain period. Conversely, the possibility of rebound hypercalcemia after several weeks of hypocalcemia treatment has been reported [17], as observed in the current case, implying the importance of regular monitoring for early detection and intervention during the treatment, regardless of drug holidays. It is crucial to successfully manage the symptoms using the aforementioned methods in patients receiving cetuximab sarotalocan, as well as treatments with other anti-EGFR monoclonal antibodies.

This case report has several limitations owing to clinical practice. First, several data values were unavailable. The variation in serum electrolytes in the case of data missing, given the variation in the other treatment cycles, may have resulted in a discrepancy from the true results. Second, the renal excretion of electrolytes was not assessed. Third, genetic backgrounds were not evaluated. Consequently, our findings should be interpreted with consideration of these limitations.

In conclusion, we report temporary independent hypocalcemia without hypomagnesemia in a patient with hypoparathyroidism who underwent NIR-PIT using cetuximab sarotalocan. Regular evaluation of serum electrolyte levels is crucial for early and appropriate symptom management in patients receiving this treatment.

## Supplementary Information

Supplementary Material 1.

## Data Availability

No datasets were generated or analysed during the current study.

## Abbreviations

HNSCC: Head and neck squamous cell carcinoma
NIR-PIT: Near-infrared photoimmunotherapy
IR700: IRDye700Dx
EGFR: Epidermal growth factor receptor
TRPM: Transient receptor potential subfamily melastatin
