# Supplementary material for: Temporary hypocalcemia induced by cetuximab sarotalocan without hypomagnesemia in a patient with hypoparathyroidism: a novel case report
Source: J Pharm Health Care Sci. 2025 Jul 4;11:58. doi: 10.1186/s40780-025-00465-y (PMC12228235; doi:10.1186/s40780-025-00465-y)
Supplement: Supplementary file 1 — Supplementary Material 1. [file 40780_2025_465_MOESM1_ESM.docx]

# **Supplemental Table 1. Naranjo Adverse Reaction Probability Scale assessment of severe hypocalcemia and cetuximab sarotalocan**

| **Question** | Yes | No | Do not know | Score |
| --- | --- | --- | --- | --- |
| 1. Are there previous conclusive reports on this reaction to the drug? | +1 | 0 | 0 | +1 |
| 1. Did the adverse event appear after the suspected drug was administered? | +2 | -1 | 0 | +2 |
| 1. Did the adverse reaction improve when the drug was discontinued or a specific antagonist was administered? | +1 | 0 | 0 | +1 |
| 1. Did the adverse reaction reappear when the drug was readministered? | +2 | -1 | 0 | +2 |
| 1. Are there alternative causes (other than the drug) that could have, on their own, caused the reaction? | -1 | +2 | 0 | -1 |
| 1. Did the reaction reappear when a placebo was given? | -1 | +1 | 0 | 0 |
| 1. Was the drug detected in blood (or other fluids) in concentrations known to be toxic? | +1 | 0 | 0 | 0 |
| 1. Was the reaction more severe when the dose was increased, or less severe when the dose was decreased? | +1 | 0 | 0 | 0 |
| 1. Did the patient have a similar reaction to the same or similar drugs in any previous exposure? | +1 | 0 | 0 | 0 |
| 1. Was the adverse event confirmed by any objective evidence? | +1 | 0 | 0 | +1 |
| Total score | | | | +6 |
